# Real-time Prescription Surveillance and its Application to Monitoring Seasonal Influenza Activity in Japan

**DOI:** 10.2196/jmir.1881

**Published:** 2012-01-16

**Authors:** Tamie Sugawara, Yasushi Ohkusa, Yoko Ibuka, Hirokazu Kawanohara, Kiyosu Taniguchi, Nobuhiko Okabe

**Affiliations:** ^1^National Institute of Infectious DiseasesInfectious Disease Surveillance CenterTokyoJapan; ^2^Hitotsubashi UniversityTokyoJapan; ^3^EM Systems Co., LtdTokyoJapan

**Keywords:** Surveillance, influenza, real-time surveillance, prescriptions, pharmacy, anti-influenza virus, automatic surveillance, early response

## Abstract

**Background:**

Real-time surveillance is fundamental for effective control of disease outbreaks, but the official sentinel surveillance in Japan collects information related to disease activity only weekly and updates it with a 1-week time lag.

**Objective:**

To report on a prescription surveillance system using electronic records related to prescription drugs that was started in 2008 in Japan, and to evaluate the surveillance system for monitoring influenza activity during the 2009–2010 and 2010–2011 influenza seasons.

**Methods:**

We developed an automatic surveillance system using electronic records of prescription drug purchases collected from 5275 pharmacies through the application service provider’s medical claims service. We then applied the system to monitoring influenza activity during the 2009–2010 and 2010–2011 influenza seasons. The surveillance system collected information related to drugs and patients directly and automatically from the electronic prescription record system, and estimated the number of influenza cases based on the number of prescriptions of anti-influenza virus medication. Then it shared the information related to influenza activity through the Internet with the public on a daily basis.

**Results:**

During the 2009–2010 influenza season, the number of influenza patients estimated by the prescription surveillance system between the 28th week of 2009 and the 12th week of 2010 was 9,234,289. In the 2010–2011 influenza season, the number of influenza patients between the 36th week of 2010 and the 12th week of 2011 was 7,153,437. The estimated number of influenza cases was highly correlated with that predicted by the official sentinel surveillance (r = .992, P < .001 for 2009–2010; r = .972, P < .001 for 2010–2011), indicating that the prescription surveillance system produced a good approximation of activity patterns.

**Conclusions:**

Our prescription surveillance system presents great potential for monitoring influenza activity and for providing early detection of infectious disease outbreaks.

## Introduction

In Japan, the official sentinel surveillance reports the number of influenza patients per health care provider after collecting information from approximately 5000 clinics and hospitals. The intensity of influenza activity is assessed according to the number of influenza patients per clinic or hospital. Influenza is regarded as highly active if the ratio exceeds 1. In 2009, the number of patients per clinic or hospital approached 1 in the 32nd week, earlier than in any of the preceding 10 years, mainly because of the influenza pandemic A (H1N1), which started in April 2009 [1]. Accordingly, the vast majority of the reported cases were H1N1 novel influenza [1]. The number of influenza patients per health care provider declined below 1 in the 13th week of 2010. The total number of weeks during which influenza was highly active was 29, a longer active period than in any of the prior 10 years. In 2010, the reported number of influenza patients per clinic or hospital exceeded 1 in the 50th week [2]; a second peak week was detected in March 2011. Because of these irregular patterns of influenza activity, it is necessary that both policy makers and clinicians follow influenza activity closely to implement effective control of an influenza outbreak throughout the year.

Syndromic surveillance is a useful tool for seasonal influenza monitoring [3]. In Japan, the official sentinel surveillance of infectious diseases is implemented by the National Institute of Infectious Diseases. It reports the estimated number of influenza patients weekly as the *Infectious Diseases Weekly Report* [2]. The official sentinel surveillance collects the number of influenza cases from approximately 5000 hospitals and clinics all over the country and then estimates the number of influenza patients based on the reported cases [4]. The entire process of collecting information from health care providers, estimating the number of clinical influenza cases, and reporting them to the public usually takes 7–10 days. Furthermore, the cases are reported by health care providers as a weekly aggregate number. Some diseases spread rapidly, and the weekly aggregates might not provide sufficiently detailed information reflecting the complete character of disease activity. In addition, the official sentinel surveillance updates influenza activity less frequently during major holidays. In Japan, seasonal influenza activity usually starts to become active during the New Year holidays. Constant monitoring and reporting of activity during that period is necessary.

Syndromic surveillance is in widespread use for monitoring diseases, but usage of prescription drug sales as a source of information is fairly limited. In the United States, the most common source of syndromic surveillance reported by health officials is emergency department visits (84%), followed by outpatient clinic visits (49%) and over-the-counter medication sales (44%); less than 10% of health departments reported prescription medications as a source [3]. In the context of influenza, emergency department surveillance is used to monitor the impact of influenza by age [5]. For more rapid feedback, the Web recently has become a powerful tool for syndromic surveillance [6]. For example, health surveillance using a Web-based self-reporting daily questionnaire is applied to monitor influenza activities [7]. Google Flu Trends, a Web-based surveillance, tracks the rate of influenza using query logs [8]. In addition to monitoring disease activities, syndromic surveillance helps monitor bioterrorism-related disease [9] or health consequences of natural events [10].

Real-time information related to influenza activity is fundamentally important for better preparation of countermeasures against a sudden increase of influenza activity. Therefore, daily updates of influenza activity are indispensable for improved understanding and control of an influenza epidemic. We developed an automatic real-time prescription surveillance system with the collaboration of EM Systems Co. Ltd. (Tokyo, Japan) to provide timely information related to a disease outbreak. We applied the surveillance system to monitor influenza activity during the 2009–2010 and 2010–2011 influenza seasons to examine the magnitude and trajectory of an outbreak more closely and to share that information with public health authorities, as well as participating pharmacies.

We used prescription drug purchase data for surveillance of influenza activity for three reasons. First, prescribing anti-influenza drugs such as oseltamivir or zanamivir is a common clinical practice for diagnosed influenza cases in Japan. Japan has the highest annual level of oseltamivir usage in the world [11]. Therefore, prescription drugs can serve as a good indicator of the overall number of influenza patients. Physicians often perform rapid influenza diagnostic tests on patients who have a fever or report other influenza-like symptoms. If the test result is positive or, alternatively, if the physician clinically diagnoses influenza even when the test result is negative, then anti-influenza drugs are often prescribed. This contrasts to practices in some other developed countries, where anti-influenza drugs are recommended for those who are at high risk [12-14] or who have severe conditions from influenza infections [13,14]. In such circumstances, surveillance of prescriptions of anti-influenza drugs would trace influenza patients with severe symptoms [15].

Second, many pharmacies have adopted the electronic prescription record system (EPRS), which enables automatic, continuous, and constant information collection, and real-time analysis of prescriptions and patients. In Japan, the utilization rate of the EPRS among pharmacies was 99.0% in 2009 [16]. Japan also has a high rate of outpatient or office-based clinician visits in cases where people feel ill [17], partly because of the universal health insurance system. Therefore, one might infer that the number of influenza patients collected through the EPRS would closely approximate the number of symptomatic influenza patients.

Third, in contrast to the United States or Taiwan [18], in Japan electronic medical record (EMR) systems are not yet well established. In the United States, surveillance for influenza activity is based on data on outpatient visits along with data related to sales of over-the-counter drugs, school absenteeism, and ambulatory care encounters [3,9,19-21]. Surveillance for influenza activity using the EMR has been intensively discussed and widely applied [22-24]. By contrast, the Survey of Medical Institutions by the Ministry of Health, Labour and Welfare in Japan showed that the share of health care providers using EMRs was just over 10% in 2008, or 948 hospitals (10.8% of all hospitals) and 12,939 clinics (13.1% of all clinics) [25].

 We developed the surveillance system to collect the number of prescriptions together with patients’ characteristics from the EPRS automatically, to analyze the data simultaneously to estimate the number of influenza cases, and then to provide real-time information of influenza activity to health care providers and policy makers. The system was tested for a limited time at the G8 Summit meeting in Toyako, Hokkaido in July 2008 for 1 month [26]. The present report summarizes details of our prescription surveillance system and presents an evaluation of its performance in the first two influenza seasons, those of 2009–2010 and 2010–2011, since the start of the nationwide operation of the system. The evaluation of surveillance performance, particularly outbreak detection performance, is challenging and few studies conduct such analyses [27]. A study showed that weekly variation in visits for lower respiratory tract infections approximated the national mortality data for pneumonia and influenza [28]. Similarly, our retrospective evaluation analyzed how closely the estimates of influenza cases followed the trajectory of influenza epidemics reported by two other sources.

## Methods

### Prescription Surveillance

**Figure 1:**
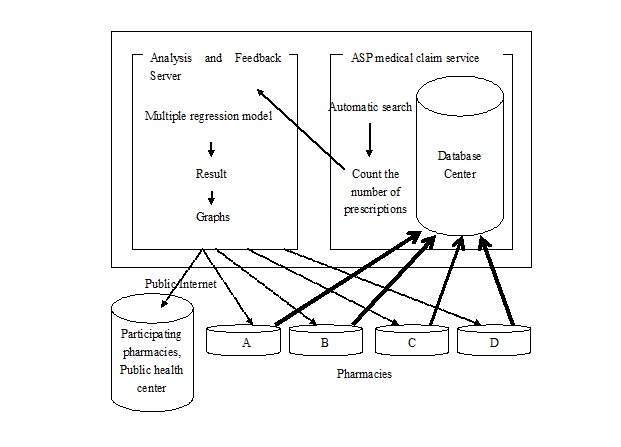
Prescription surveillance. Pharmacies A–D use the application service provider’s (ASP) medical claims service. All data are stored in a central database. The surveillance system automatically counts oseltamivir, zanamivir, and laninamivir prescriptions at the data center. The information is analyzed using multiple regression models. The results are presented as figures and tables and feedback to participating pharmacies as well as public health authorities.

We started collecting and analyzing data related to prescriptions automatically through the application service provider of the EPRS in April 2009 (Figure 1 [29]). As of March 2011, the number of participating pharmacies was 5275. In the application service provider, data related to prescriptions from all participating pharmacies were collected and deposited in a single server, making the data collection secure, efficient, and nearly cost-free. Medications covered by the surveillance system included drugs for relief of fever and pain, drugs for common colds, antibiotics, and antiviral drugs including anti-influenza virus drugs and antivaricella-zoster virus drugs. The current study specifically addressed prescriptions for anti-influenza virus medication. The neuraminidase inhibitors oseltamivir, zanamivir, and laninamivir were included, but amantadine was excluded because it is not commonly prescribed for influenza in Japan.

 The original prescriptions contain information related to patients’ sociodemographic and social security information, as well as the health care providers’ information. The automatic surveillance system aggregated the number of prescriptions for each type of drug and provided tabulations by age and by geography at both national and prefectural levels. The number of influenza patients was then estimated from the aggregated number of prescriptions for anti-influenza drugs by adjusting the number of prescriptions for anti-influenza drugs with the proportion of participating pharmacies and of prescriptions purchased through pharmacies. The analysis and estimation were conducted overnight and the report of the analysis was sent automatically at 7:00 AM on the next day to the registered recipients, including participating pharmacies and public health authorities. In addition, figures showing the number of prescriptions for each type of drug and of the estimated number of patients were created and posted on the website for public access.

### Performance Evaluation

We evaluated our surveillance system from two perspectives for the 2009–2010 and 2010–2011 influenza seasons. First, we compared the estimated number of influenza patients with the estimates provided by the official sentinel surveillance [2]. The official sentinel surveillance estimates the number of influenza patients based on the number of influenza patients reported by 5000 health care providers, including 3000 pediatricians, in Japan. We chose the evaluation period to include the period when influenza activity was high for the 2009–2010 influenza season. The epidemiological threshold of seasonal influenza activity is determined by the number of influenza patients per hospital or clinic. If the ratio is equal to or greater than 1 based on the official sentinel surveillance, activity is *high* by the definition that is accepted and widely used throughout Japan [2]. This corresponds to the period between the 28th week of 2009 (the week starting on July 6, 2009) and the 12th week of 2010 (the week starting on March 21, 2010) for the 2009–2010 influenza season. For the 2010–2011 season, the performance was evaluated between the 36th week of 2010 (the week starting on September 6, 2010) and the 12th week of 2011 (the week starting on March 21, 2011). Second, for the 2009–2010 influenza season, we also compared our estimates with the number of influenza patients estimated by the Gifu Medical Association, where the total number of influenza patients in the prefecture was calculated and reported publicly [29]. The number of influenza patients in Gifu Prefecture was surveyed during November 16–22, 2009 by the local public health authority as a response to the A/H1N1 influenza pandemic. A survey questionnaire asking for the number of influenza patients who visited health care providers was sent to all hospitals and clinics located within the prefecture (total of 1677 health providers); 1033 providers responded to the survey (response rate 61.6%) [29].

 The Internal Review Board at the National Institute of Infectious Diseases approved the current study (approval number 57, “Development and application of real-time surveillance system to monitor syndromic and symptomatic cases using electronic record system”).

## Results

For the 2009–2010 influenza season, the total number of influenza patients estimated by the prescription surveillance system between the 28th week of 2009 and the 12th week of 2010 was 9,234,289 (Table 1). The largest number of influenza patients, 234,519, was reported on November 24, 2009. For the 2010–2011 influenza season, the number of influenza patients between the 36th week of 2010 and the 12th week of 2011 was 7,153,437 (Table 1). The largest number of influenza patients, 230,288, was reported on January 24, 2011. The official sentinel surveillance estimated the total number of patients for the same periods as 20,660,000 (95% confidence interval 20,460,000–20,860,000) for the 2009–2010 and 13,680,000 (95% confidence interval 13,350,000–14,010,000) for the 2010–2011 influenza seasons [2], indicating that the sentinel estimates were approximately double our estimates.

**Table 1:** Number of influenza cases estimated by the prescription surveillance, the official sentinel surveillance, and the Gifu Medical Association in Gifu Prefecture, 2009–2010 and 2010–2011 influenza seasons^a^

	2009–2010 influenza season: July 6, 2009–March 28, 2010 (28th week 2009–12th week 2010)	2010–2011 influenza season: September 6, 2010–March 27, 2011 (36th week 2010–12th week 2011)
Estimate by the prescription surveillance	9,234,289	7,153,437
Estimate by the official sentinel surveillance	20,660,000	13,680,000
Adjusted estimation by the survey in Gifu Prefecture	9,931,200	Not applicable^b^

^a^ Sources: the official sentinel surveillance [2]; Kawai et al [29].

^b^ Adjusted estimation by the survey in Gifu Prefecture is shown only for the 2009–2010 influenza season because the data are available only for that year.

**Figure 2:**
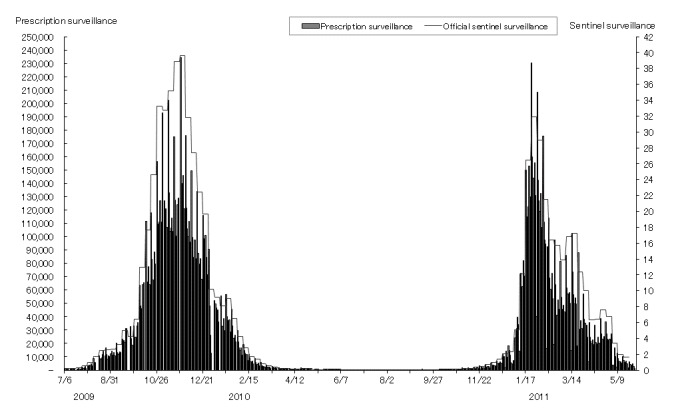
Number of influenza cases, 2009–2011, estimated by the prescription surveillance and reported by the official sentinel surveillance. The estimated number of influenza cases by prescription surveillance was calculated based on the number of oseltamivir, zanamivir, and laninamivir prescriptions adjusted by the proportion of participating pharmacies and extramural dispensing percentage. See text for details. The reported number by the official sentinel surveillance shows the number of influenza patients per clinic or hospital, calculated with the reported number of influenza patients from 5000 sentinel clinics and hospitals.

 Pearson correlation coefficient (*r*) of time-series data on influenza patients between our estimates and the official sentinel estimate was .992 (*P* < .001) for the 2009–2010 influenza season, and .972 (*P* < .001) for the 2010–2011 influenza season (see Figure 2). A similar analysis was conducted at the prefecture level. The correlation was.950 or greater in 33 prefectures, .900–.949 in 5 prefectures, and .770–.899 in 8 prefectures. The correlation was the lowest in Akita Prefecture (*r* = .689).

 The estimated number of influenza cases in the 2009–2010 influenza season was also compared with that ascertained from the survey of the number of influenza patients at all clinics and hospitals conducted in Gifu Prefecture. The estimated number from the survey collection in the prefecture based on the prescription surveillance was 127,568, whereas the number of influenza cases reported by the survey conducted by Gifu Medical Association was 132,474. The official sentinel surveillance estimated the number as 277,890.

## Discussion

Our analyses showed that the time-series pattern of influenza activity reported by the prescription surveillance system in the first two influenza seasons was highly correlated with the pattern reported by the official sentinel surveillance, showing that pharmacy surveillance can be a good indicator of influenza activity in Japan. Although the estimated number of influenza patients was double that of the official sentinel surveillance, it was close to the estimate by Gifu Prefecture, where the total number of influenza patients was collected in a survey.

The significance of our prescription surveillance is threefold. First, the syndromic surveillance system collected, analyzed, and reported data related to influenza patients simultaneously. Therefore, clinicians and policy makers were able to obtain the estimated number of influenza patients of the previous day. This meant that the estimates were available 1 week ahead of those reported by the official sentinel surveillance, enabling predictions of influenza activity for the immediately following week. This was particularly important at the outset of a seasonal epidemic, when the trajectory of a quickly spreading disease would have changed. Though the Google Flu Trends tool, another real-time surveillance, has been shown to perform well in the United States [8] and European countries [30], the results may be sensitive to variations in patients’ behavior across countries.

Second, our prescription surveillance was national and observed regional variations in influenza activity at the prefecture level, although the precision of surveillance varied somewhat between prefectures. This provided helpful information to public health services to plan for the allocation of medical, pharmaceutical, and human resources for influenza control, shifting limited resources to the most affected regions.

Third, our surveillance runs constantly, maintaining the method of counting and estimating influenza cases at all times, and thus we were able to obtain the complete trajectory of the influenza pandemic in the 2009–2010 season. Initially during the pandemic, the law required hospitals and clinics to report all influenza cases, but that practice was terminated on July 24, 2009, after which activity was tracked only by the official sentinel surveillance.

Our surveillance system also promises great potential for future application to the early detection of an infectious disease outbreak or bioterrorism attack, which could happen potentially anywhere at any time. When we started operating a prescription surveillance system in 2009, all other surveillance systems running in Japan covered only specific regions of the country for practical reasons [31]. Furthermore, because influenza outbreaks do not necessarily occur during winter, the time that is covered by the sentinel surveillance, continuous monitoring of influenza activity is necessary to detect outbreaks early in their course. Our automatic prescription surveillance system uses the same standard for detection of a disease outbreak and runs continuously, providing an important complementary role in support of existing surveillance systems in Japan.

If EMRs were widely kept, then information related to influenza patients could be collected even faster and possibly more accurately. However, the share of health care providers that have adopted the EMR system was slightly above 10%. Under such circumstances, purchases of anti-influenza drugs can serve as an alternative indicator of influenza activity.

Limitations to this study exist. First, the total number of influenza cases was estimated as almost half of the estimate based on the official sentinel surveillance, although it approximated estimates based on a survey collecting the total number of influenza cases in Gifu Prefecture. One reason for this gap might lie in the choice of health care providers participating in the official sentinel surveillance. The sentinel health care providers have, on average, a larger number of patients than others, potentially resulting in an overestimation of the overall number of influenza patients. Second, anti-influenza drugs are also prescribed for prophylaxis in addition to treatment, which might engender overestimation of the total number of influenza cases. However, in Japan the preventive usage of oseltamivir is limited to household members of influenza patients who are 65 years or older or who are high-risk individuals [32]. Third, the prophylactic usage of anti-influenza drugs for health care providers and for the public was most intensive at the beginning of the H1N1 pandemic outbreak. We did not include those prescriptions in our surveillance data because they were not prescribed through health care providers. Fourth, 60% of the prescriptions were purchased through pharmacies in 2008. The other prescriptions were purchased directly through health care providers and were not included in our surveillance [33]. This is still much higher than the rate of adoption of the EMR system in hospitals and clinics. Fifth, the participation rate of pharmacies is low, particularly in certain areas. If the number of participating pharmacies were increased, then estimating influenza cases would be possible even for smaller geographical units.

Despite these limitations, pharmacy surveillance provided an approximation of the trend of influenza activity in the first two influenza seasons after the start of its nationwide operation. It provided both clinicians and policy makers with helpful real-time information related to influenza activity. Our pharmacy surveillance system has great potential for detection as well as for monitoring of infectious disease outbreaks in the population and in cases of significant political or cultural events.

## Abbreviations

EMR: electronic medical record
EPRS: electronic prescription record system

## References

[1] Shimada T, Gu Y, Kamiya H, Komiya N, Odaira F, Sunagawa T, Takahashi H, Toyokawa T, Tsuchihashi Y, Yasui Y, Tada Y, Okabe N (2009). Epidemiology of influenza A(H1N1)v virus infection in Japan, May-June 2009. Euro Surveill.

[2] (2011). National Institute of Infectious Diseases.

[3] Buehler JW, Sonricker A, Paladini M, Sope P, Mostashar F (2008). Syndromic surveillance practice in the United States: findings from a survey of state, territorial, and selected local health departments. Adv Dis Surveill.

[4] Taniguchi K, Hashimoto S, Kawado M, Murakami Y, Izumida M, Ohta A, Tada Y, Shigematsu M, Yasui Y, Nagai M (2007). Overview of infectious disease surveillance system in Japan, 1999-2005. J Epidemiol.

[5] Olson DR, Heffernan RT, Paladini M, Konty K, Weiss D, Mostashari F (2007). Monitoring the impact of influenza by age: emergency department fever and respiratory complaint surveillance in New York City. PLoS Med.

[6] Brownstein JS, Freifeld CC, Reis BY, Mandl KD (2008). Surveillance Sans Frontières: Internet-based emerging infectious disease intelligence and the HealthMap project. PLoS Med.

[7] Sugiura H, Ohkusa Y, Akahane M, Sano T, Okabe N, Imamura T (2011). Development of a web-based survey for monitoring daily health and its application in an epidemiological survey. J Med Internet Res.

[8] Ginsberg J, Mohebbi MH, Patel RS, Brammer L, Smolinski MS, Brilliant L (2009). Detecting influenza epidemics using search engine query data. Nature.

[9] Buehler JW, Berkelman RL, Hartley DM, Peters CJ (2003). Syndromic surveillance and bioterrorism-related epidemics. Emerg Infect Dis.

[10] Elliot AJ, Singh N, Loveridge P, Harcourt S, Smith S, Pnaiser R, Kavanagh K, Robertson C, Ramsay CN, McMenamin J, Kibble A, Murray V, Ibbotson S, Catchpole M, McCloskey B, Smith GE (2010). Syndromic surveillance to assess the potential public health impact of the Icelandic volcanic ash plume across the United Kingdom, April 2010. Euro Surveill.

[11] Ujike M, Shimabukuro K, Mochizuki K, Obuchi M, Kageyama T, Shirakura M, Kishida N, Yamashita K, Horikawa H, Kato Y, Fujita N, Tashiro M, Odagiri T, Working Group for Influenza Virus Surveillance in Japan (2010). Oseltamivir-resistant influenza viruses A (H1N1) during 2007-2009 influenza seasons, Japan. Emerg Infect Dis.

[12] (2009). National Institute for Health and Clinical Excellence.

[13] (2011). Centers for Disease Control and Prevention.

[14] Harper SA, Bradley JS, Englund JA, File TM, Gravenstein S, Hayden FG, McGeer AJ, Neuzil KM, Pavia AT, Tapper ML, Uyeki TM, Zimmerman RK, Expert Panel of the Infectious Diseases Society of America (2009). Seasonal influenza in adults and children--diagnosis, treatment, chemoprophylaxis, and institutional outbreak management: clinical practice guidelines of the Infectious Diseases Society of America. Clin Infect Dis.

[15] Sugawara T, Ohkusa Y, Kawanohara H, Taniguchi K, Okabe N (2011). [The real-time pharmacy surveillance and its estimation of patients in 2009 influenza A (H1N1)]. Kansenshogaku Zasshi.

[16] (2010). Ministry of Health, Labour and Welfare, Japan.

[17] Sugawara T, Ohkusa Y, Kondo M, Honda Y, Okubo I (2005). [Research on choices of people with mild symptoms of common cold between consulting physicians and taking OTC (over-the-counter) medicine using a hypothetical question method]. Nihon Koshu Eisei Zasshi.

[18] Wu TS, Shih FY, Yen MY, Wu JS, Lu SW, Chang KC, Hsiung C, Chou JH, Chu YT, Chang H, Chiu CH, Tsui FC, Wagner MM, Su IJ, King CC (2008). Establishing a nationwide emergency department-based syndromic surveillance system for better public health responses in Taiwan. BMC Public Health.

[19] Lazarus R, Kleinman K, Dashevsky I, Adams C, Kludt P, DeMaria A, Platt R (2002). Use of automated ambulatory-care encounter records for detection of acute illness clusters, including potential bioterrorism events. Emerg Infect Dis.

[20] Henning KJ (2004). What is syndromic surveillance?. MMWR Morb Mortal Wkly Rep.

[21] Ohkusa Y, Sugiura H, Sugawara T, Taniguchi K, Okabe N (2006). [Symptoms of outpatients as data for syndromic surveillance]. Kansenshogaku Zasshi.

[22] South BR, South BR, Chapman WW, Chapman W, Delisle S, Shen S, Kalp E, Perl T, Samore MH, Gundlapalli AV (2008). Optimizing A syndromic surveillance text classifier for influenza-like illness: Does document source matter?. AMIA Annu Symp Proc.

[23] Gundlapalli AV, Olson J, Smith SP, Baza M, Hausam RR, Eutropius LJ, Pestotnik SL, Duncan K, Staggers N, Pincetl P, Samore MH (2007). Hospital electronic medical record-based public health surveillance system deployed during the 2002 Winter Olympic Games. Am J Infect Control.

[24] Lewis MD, Pavlin JA, Mansfield JL, O'Brien S, Boomsma LG, Elbert Y, Kelley PW (2002). Disease outbreak detection system using syndromic data in the greater Washington DC area. Am J Prev Med.

[25] (2011). Ministry of Health, Labour and Welfare, Japan.

[26] Ohkusa Y, Yamaguchi R, Sugiura H, Sugawara T, Yoshida M, Shimada C, Hori N, Sugishita Y, Yasui Y, Sunagawa T, Matsui T, Taniguchi K, Tada Y, Taya K, Imamura T, Okabe N (2009). [2008 G8 Hokkaido Toyako Summit Meeting Syndrome Surveillance]. Kansenshogaku Zasshi.

[27] Mandl KD, Overhage JM, Wagner MM, Lober WB, Sebastiani P, Mostashari F, Pavlin JA, Gesteland PH, Treadwell T, Koski E, Hutwagner L, Buckeridge DL, Aller RD, Grannis S (2004). Implementing syndromic surveillance: a practical guide informed by the early experience. J Am Med Inform Assoc.

[28] Lazarus R, Kleinman K, Dashevsky I, DeMaria A, Platt R (2001). Using automated medical records for rapid identification of illness syndromes (syndromic surveillance): the example of lower respiratory infection. BMC Public Health.

[29] Kawai N, Kawade Y, Kobayashi H, Okada S, Higuchi Y, Kawaji H, Ohkusa Y (2010). Analysis of influenza activity during 2009 influenza pandemic A (H1N1) using real-time infectious disease surveillance in Gifu prefecture [in Japanese]. Syukan Nihon Iji Shinpou.

[30] Valdivia A, Lopez-Alcalde J, Vicente M, Pichiule M, Ruiz M, Ordobas M (2010). Monitoring influenza activity in Europe with Google Flu Trends: comparison with the findings of sentinel physician networks - results for 2009-10. Euro Surveill.

[31] Ohkusa Y, Shigematsu M, Taniguchi K, Okabe N (2005). Experimental surveillance using data on sales of over-the-counter medications--Japan, November 2003-April 2004. MMWR Morb Mortal Wkly Rep.

[32] (2011). Chugai Pharmaceutical Co. Ltd.

[33] (2008). Ministry of Health, Labour and Welfare, Japan.

